# Hepatocellular carcinoma associated microRNA expression signature: integrated bioinformatics analysis, experimental validation and clinical significance

**DOI:** 10.18632/oncotarget.4437

**Published:** 2015-06-30

**Authors:** Ke-Qing Shi, Zhuo Lin, Xiang-Jian Chen, Mei Song, Yu-Qun Wang, Yi-Jing Cai, Nai-Bing Yang, Ming-Hua Zheng, Jin-Zhong Dong, Lei Zhang, Yong-Ping Chen

**Affiliations:** ^1^ Department of Infection and Liver Diseases, Liver Research Center, The First Affiliated Hospital of Wenzhou Medical University, Wenzhou 325000, Zhejiang Province, China; ^2^ Department of General Surgery, The First Affiliated Hospital of Wenzhou Medical University, Wenzhou 325000, Zhejiang Province, China

**Keywords:** hepatocellular carcinoma, microRNA, biomarker, signature, robust rank aggregation

## Abstract

microRNA (miRNA) expression profiles varied greatly among current studies due to different technological platforms and small sample size. Systematic and integrative analysis of published datesets that compared the miRNA expression profiles between hepatocellular carcinoma (HCC) tissue and paired adjacent noncancerous liver tissue was performed to determine candidate HCC associated miRNAs. Moreover, we further validated the confirmed miRNAs in a clinical setting using qRT-PCR and Tumor Cancer Genome Atlas (TCGA) dataset. A miRNA integrated-signature of 5 upregulated and 8 downregulated miRNAs was identified from 26 published datesets in HCC using robust rank aggregation method. qRT-PCR demonstrated that miR-93-5p, miR-224-5p, miR-221-3p and miR-21-5p was increased, whereas the expression of miR-214-3p, miR-199a-3p, miR-195-5p, miR-150-5p and miR-145-5p was decreased in the HCC tissues, which was also validated on TCGA dataset. A miRNA based score using LASSO regression model provided a high accuracy for identifying HCC tissue (AUC = 0.982): HCC risk score = 0.180E_miR-221 + 0.0262E_miR-21 - 0.007E_miR-223 - 0.185E_miR-130a. E_miR-n = Log 2 (expression of microRNA n). Furthermore, expression of 5 miRNAs (miR-222, miR-221, miR-21 miR-214 and miR-130a) correlated with pathological tumor grade. Cox regression analysis showed that miR-21 was related with 3-year survival (hazard ratio [HR]: 1.509, 95%CI: 1.079–2.112, *P* = 0.016) and 5-year survival (HR: 1.416, 95%CI: 1.057–1.897, *P* = 0.020). However, none of the deregulated miRNAs was related with microscopic vascular invasion. This study provides a basis for further clinical application of miRNAs in HCC.

## INTRODUCTION

Hepatocellular carcinoma (HCC) is one of the most common cancers worldwide and the third most common cause of cancer-related death worldwide [1]. The prognosis of HCC is very poor, with a median survival of 6 to 20 months and less than 5% of symptomatic patients surviving more than 2 years. Identification of new biomarkers for the early detection of HCC is critical for the patients to receive proper therapeutic treatment as early as possible.

MicroRNA (miRNA), as a class of short noncoding RNA molecules, controlling approximate one third of the protein-coding genes by posttranscriptional regulation of gene expression can directly or indirectly affect almost all cellular pathways [2]. Recent years, the number of miRNA profiling studies has increased rapidly. Specific miRNA aberrations involved in cancer development and progression have been identified by high-throughput technologies across different normal and cancer tissues [3–5]. Therefore, many miRNAs are proposed as promising biomarkers for early detection of HCC and accurate predictions of prognosis, as well as targets for treatment [6, 7]. Unfortunately, the common drawback of miRNA expression profiling studies is a lack of agreement due to many factors including application of different technological platforms, small sample size, inconsistent annotation, ongoing discovery of novel miRNAs, and use of different methods for data processing and analysis [8–10].

To overcome these limitations, we need to integrate their results in an unbiased manner. Robust rank aggregation (RRA) approach has been specifically designed for comparison of several ranked gene lists [11]. The tool looks at how each item is positioned in the ranked lists and compares this to the baseline case where all the preference lists are randomly shuffled. As a result, a *P*-value would be assigned for all items, showing how much better it was positioned in the ranked lists than expected by chance. This *P*-value is used both for re-ranking the items and deciding their significance. RRA is a suitable and effective solution for identification of statistically significant miRNA integrated-signature and is particularly useful when input experiments are performed by different technological platforms cover different sets of genes and full rankings of miRNAs are not available.

Herein, we would use this integrated bioinformatics approach to obtain a consistent miRNA expression signature as well as novel miRNA-regulated molecular pathways that contribute to the pathogenesis of HCC, which would help to prioritize the putative targets for further experimental studies of HCC development. Moreover, we further validated the most dysregulated miRNAs in a clinical setting.

## RESULTS

### Characteristics of the datasets

According to the inclusion criteria, 26 independent full-text studies retrieved from public databases (GEO, ISI Web of Science, and ArrayExpress) were used to build the 26 HCC miRNA expression profiling datasets. Of the 26 HCC miRNA expression profiling datasets, 1 profiling datasets were re-analyzed in GEO DataSets [12], while the others were extracted from the published studies directly. The description of the studies was provided in Supplementary Table 1.

**Figure 1:**
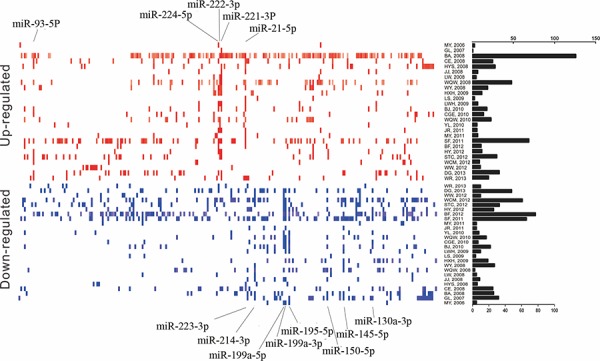
Distribution of miRNA alterations in HCC as reported in 26 miRNA profiles datasets Upregulated and downregulated miRNAs were shown as short red and blue vertical bars, respectively. miRNAs are aligned according to miRBase release 21. The number of miRNAs in each study is graphically depicted on the right. The positions of HCC imtegrated-signature miRNAs were marked. HCC, hepatocellular carcinoma; miRNA, microRNA.

Our integrated dataset included a total of 1250 pairs tumor and adjacent noncancerous tissues. The number of samples investigated ranged from 8 to 241 pairs (median 21) across the studies. Various microarray platforms were used in the studies (either commercial or custom) and the median number of miRNA probes assayed was 384 (ranging from 114 to 208818). Distribution of HCC-specific miRNA alterations as reported by primary studies was shown in Figure 1, which reflected the expansion of studied miRnome from 2006 to 2013.

In total, 278 significantly up-regulated miRNAs and 231 significantly down-regulated miRNAs were reported in at least one study, respectively. In addition, 39 discordant alteration miRNAs were reported, indicating that they both up-regulated and down-regulated across the different studies. The number of significantly dysregulated miRNAs varies greatly across the studies. At least two upregulated or downregulated miRNAs were reported in each study, with the exception of GL, 2007 [13], in which only one up-regulated miRNA was reported. The deregulated miRNA lists varies across the studies (Supplementary Figure 1). Finally, the rank matrixes of normalized upregulated and downregulated miRNAs lists were separate constructed (Table 1).

**Table 1:** Hepatocellular carcinoma associated microRNAs

microRNA	Chromosome	Permutation *p*-value	Corrected *p*-value	No. of Studies	Seed family	microRNA Cluster
Upregulated						
miR-93-5p	7q22.1	1.55E–10	3.23E–05	12	miR-17/17-5p/20ab/20b-5p/93/106ab/427/518a-3p/519d	miR-25/miR-93/miR-106
miR-224-5p	Xq28	5.85E–14	1.22E–08	12	miR-224	miR-224/miR-452
miR-222-3p	Xp11.3	5.81E–14	1.21E–08	12	miR-221/222/222ab/1928	miR-221/miR-222
miR-221-3p	Xp11.3	7.10E–17	1.48E–11	16	miR-221/222/222ab/1928	miR-221/miR-222
miR-21-5p	17q23.1	3.65E–12	7.63E–07	15	miR-21/miR-590-5p	-
Downregulated						
miR-223-3p	Xq12	2.59E–09	5.40E–04	9	miR-223	-
miR-214-3p	1q24.3	1.01E–13	2.11E–08	13	miR-214/761/3619-5p	miR-199/miR-214
miR-199a-5p	19p13.2/1q24.3	2.95E–19	6.17E–14	14	miR-199a-5p	-
miR-199a-3p	19p13.2/1q24.3	1.20E–16	2.52E–11	15	miR-199ab-3p/3129-5p	-
miR-195-5p	17p13.1	1.85E–11	3.86E–06	14	miR-15abc/16/16abc/195/322/424/497/1907	miR-195/miR-497
miR-150-5p	19q13.33	2.60E–09	5.44E–04	10	miR-150/5127	-
miR-145-5p	5q32	4.90E–12	1.02E–06	13	miR-145	miR-143/miR-145
miR-130a-3p	11q12.1	3.13E–09	6.55E–04	9	miR-130ac/301ab/301b/301b-3p/454/721/4295/3666	miR-130/miR-301/miR-454

### HCC associated microRNA expression signature

A *P*-value was assigned for each miRNA using RRA. Five upregulated (miR-93-5p, miR-224-5p, miR-222-3p, miR-221-3p, and miR-21-5p) and eight downregulated (miR-223-3p, miR-214-3p, miR-199a-5p, miR-199a-3p, miR-195-5p, miR-150-5p, miR-145-5p, and miR-130a-3p) miRNAs in HCC samples compared to matched non-tumor liver tissue were identified. All integrated-signature miRNAs reached statistical significance after Bonferroni correction and were reported by at least 1/3 datasets. Among these integrated-signature miRNAs, miR-199a, was represented by both its “major” (miR-199a-3p) and “minor“ (miR-199a-5p) forms. Seven of the most significantly dysregulated miRNAs, miR-221-3p, miR-21-5p, miR-214-3p, miR-199a-5p, miR-199a-3p, miR-195-5p and miR-145-5p, were reported by 1/2 datasets (Table 1). The expression change of integrated-signature miRNAs was consistent across corresponding studies. Corrected *p*-value of integrated-signature miRNAs ranged from 6.17E-14 to 6.55E-04. Most integrated-signature miRNAs belonged to the broadly conserved seed families (conserved across most vertebrates, usually to zebrafish), while miR-224-5p was sorted as conserved seed families (conserved across most mammals, but usually not beyond placental mammals), and miR-199a-3p was from poorly conserved seed families.

A cluster of miRNAs was defined as that miRNAs were located at a distance of less than 50 kb, were transcribed in the same direction and were not separated by a transcription unit or a miRNA in the opposite orientation. Therefore, 8 integrated-signature miRNAs belong to the cluster of two or more miRNAs.

In the integrated datasets, 8 studies were focused only on hepatitis B virus (HBV) related HCC [10, 14–20], and one study included only hepatitis C virus (HCV) infection patients [21], whereas most of the studies were not focused on any specific etiology related HCC [3–9, 12, 13, 22–29]. Therefore, we reanalyzed subsets of datasets derived from HBV related HCC samples. Analysis of studies which consisted of samples with HBV infection only, showed that miR-222-3p (Bonferroni-corrected *p*-value = 1.74E-05), miR-21-5p (Bonferroni-corrected *p*-value = 4.69E-04), and miR-221-3p (Bonferroni-corrected *p*-value = 1.71E-02) reached statistical significance in upregulated gene lists. In addition, miR-199a-5p (Bonferroni-corrected *p*-value = 6.33E-05), miR-145-5p (Bonferroni-corrected *p*-value = 5.50E-03), and miR-199a-3p (Bonferroni-corrected *p*-value = 2.31E-02) were significantly downregulated in HBV related HCC.

### Experimental validation of expression of the integrated-signature miRNAs in patients with HCC and clinical significance

**Figure 2:**
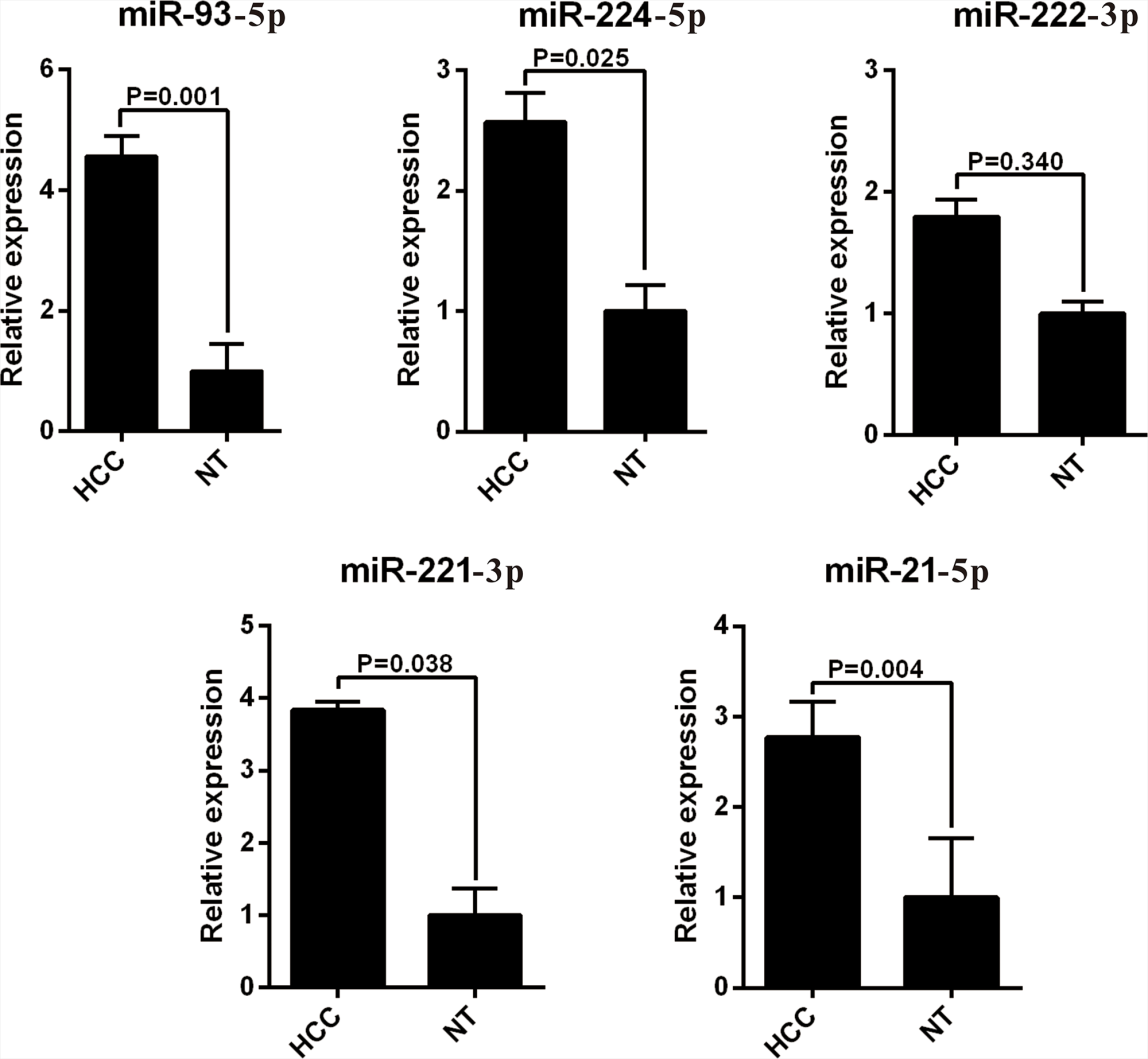
RT-PCR analysis of upregulated miRNAs expression in the HCC tissues and the adjacent noncancerous liver tissues HCC, hepatocellular carcinoma; miRNA, microRNA.

**Figure 3:**
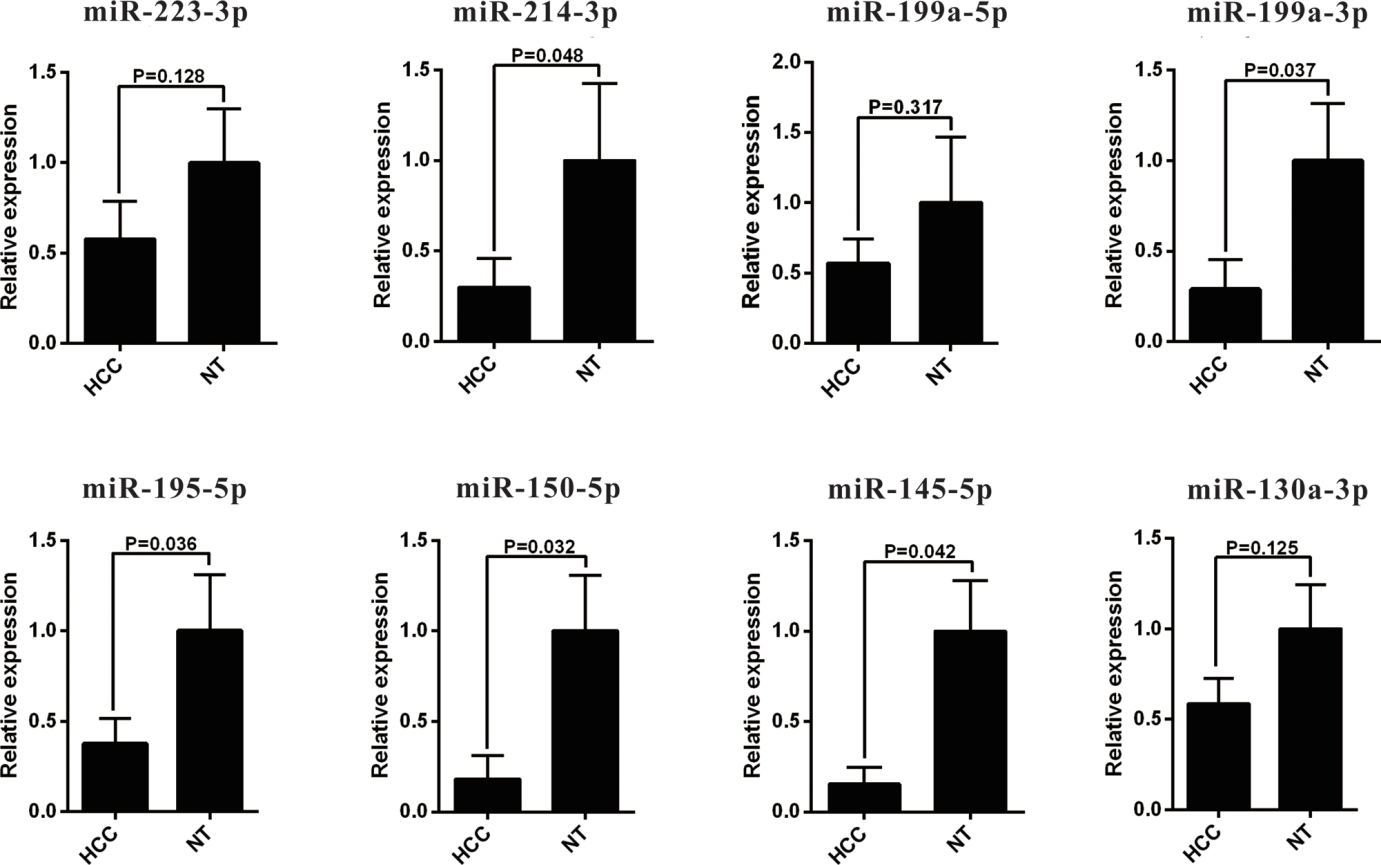
qRT-PCR analysis of downregulated miRNAs expression in the HCC tissues and the adjacent noncancerous liver tissues HCC, hepatocellular carcinoma; miRNA, microRNA.

**Figure 4:**
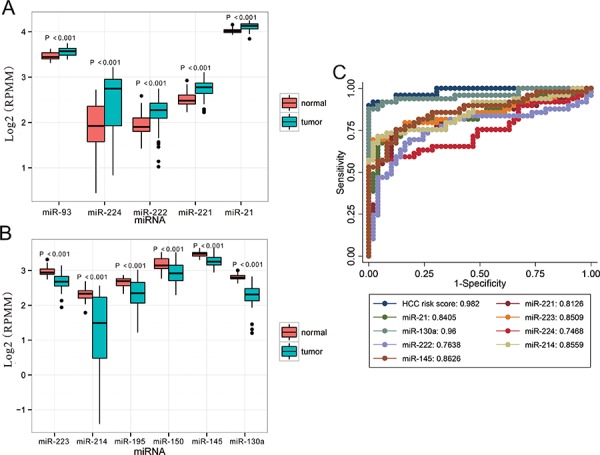
Validation of miRNAs expression in HCC on the TCGA dataset **A.** Upregulated miRNAs expression. **B.** Downregulated miRNAs expression. **C.** Performances of the miRNAs in HCC tissue classification. For boxplots, expression values of miRNAs were log2-transformed and box width was proportional to the square root of sample size in each variant. HCC risk score was built using LASSO regression model by R software: 0.180E_miR-221 + 0.0262E_miR-21 - 0.007E_miR-223 - 0.185E_miR-130a. E_miR-n = Log 2 (expression of microRNA n). HCC, hepatocellular carcinoma; miRNA, microRNA; TCGA, Tumor Cancer Genome Atlas.

The 13 most deregulated miRNAs from the integrated bioinformatics analysis were determined by qRT-PCR analysis. The results showed that the expression levels of miR-93-5p, miR-224-5p, miR-221-3p and miR-21-5p were increased more than 2 folds (Figure 2, *P* < 0.05), whereas the levels of miR-214-3p, miR-199a-3p, miR-195-5p, miR-150-5p and miR-145-5p were decreased more than 2 folds in the HCC tissues (Figure 3, *P* < 0.05). Consistent with our initial analysis, 11 miRNAs were found to be significantly dysregulated in HCC tissues in Tumor Cancer Genome Atlas (TCGA) data base (49 pairs of tumorous and adjacent nontumorous liver tissues) (Figure 4A, Figure 4B), except miR-199a-5p and miR-199a-3p which were not listed. However, the expressions changed more than 2-fold were found in miR-224-5p, miR-222-3p, miR-221-3p, miR-21-5p, miR-223-3p, miR-214-3p, miR-145-5p and miR-130a-3p. In addition, the performances of these 8 validated miRNAs in HCC tissue classification were estimated using receiver operating characteristic (ROC) curve analysis. Each miRNA had a good predictive performance. The combined miRNAs panel using LASSO regression model provided a high classification accuracy of HCC tissue (AUC = 0.982) [30, 31]: HCC risk score = 0.180E_miR-221 + 0.0262E_miR-21 - 0.007E_miR-223 - 0.185E_miR-130a (Figure 4C). E_miR-n = Log 2 (expression of microRNA n). The TCGA results showed that comparing to well-differentiated tumor grade, miR-93-5p, miR-224-5p, miR-222-3p, miR-221-3p and miR-21-5p were significantly increased, whereas miR-214-3p significantly decreased in the moderately/poorly differentiated tumor grade (Supplementary Figure 2A, 2B). However, none of the miRNAs had 2-fold changes. The Grade score combined miRNAs using LASSO regression model had a relative good performance: 0.0427E_miR-222 + 0.0030E_miR-221 + 0.0763E_miR-21 - 0.0184E_miR-214-3p + 0.0098E_miR-130a (Supplementary Figure 2C). The predictive power of the single miRNA was low. However, none of the 13 most deregulated miRNAs was related with MVI in TCGA data (Supplementary Figure 3).

**Figure 5:**
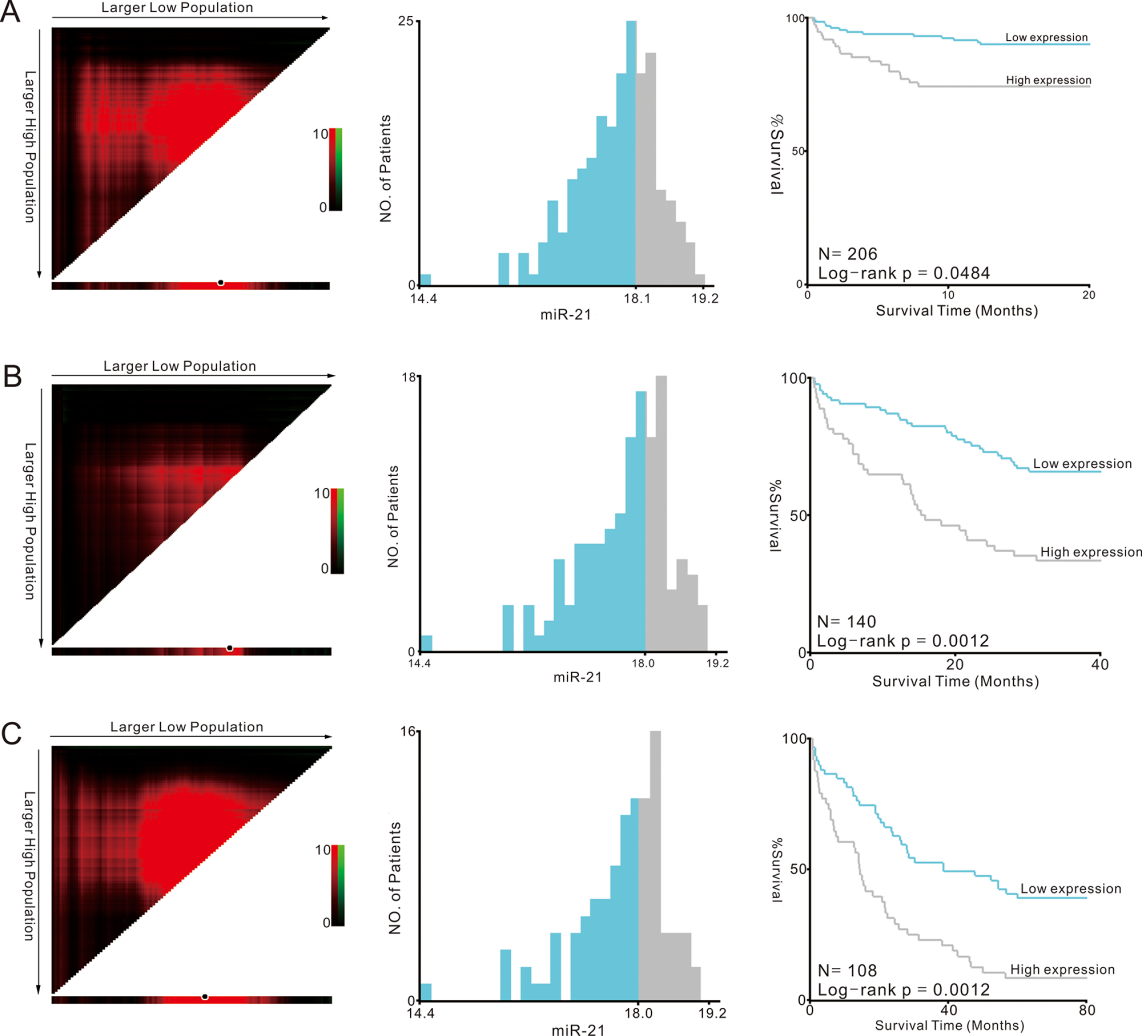
Kaplan-Meier survival analysis by X-tile plots cut-off point **A.** 1-year survival analysis; **B.** 3-year survival analysis; **C.** 5-year survival analysis X-tile plots are shown in the left panels. The plot showed the chi-squared log-rank values created when the cohort was divided into two groups. The optimal cut-point highlighted by the black circle in the left panels is shown on a histogram of the entire cohort (middle panels) and a Kaplan-Meier plot (right panels).

Furthermore, we used Cox regression analysis to build a prognostic classifier, by which only miR-21 was selected: miR-21 (hazard ratio [HR]: 1.509, 95%CI: 1.079–2.112, *P* = 0.016) for 3-year survival and miR-21 (HR: 1.416, 95%CI: 1.057–1.897, *P* = 0.020) for 5-year survival, respectively. X-tile and K-M survival analysis also showed the miR-21 could predict the clinical outcome of TCGA (Figure 5).

### Targets prediction and functional enrichment

The high-stringency target prediction for validated miRNAs was conducted. Target genes were obtained from both prediction algorithms and experimentally supported databases. The counts of predicted targets, experimentally validated targets, prediction based consensus targets, and consensus targets were summarized in Supplementary Figure 4. miR-21-5p, miR-195-5p, and miR-214-3p had highest number of consensus targets, whereas miR-199a-3p were the miRNAs with smallest number of consensus targets. In addtion, we performed enrichment analyses to elucidate the biological function of miRNA integrated-signature using target genes. Finally, 72 Panther pathways, 143 KEGG pathways, and 857 GO processes were enriched with the miRNAs targets.

**Figure 6:**
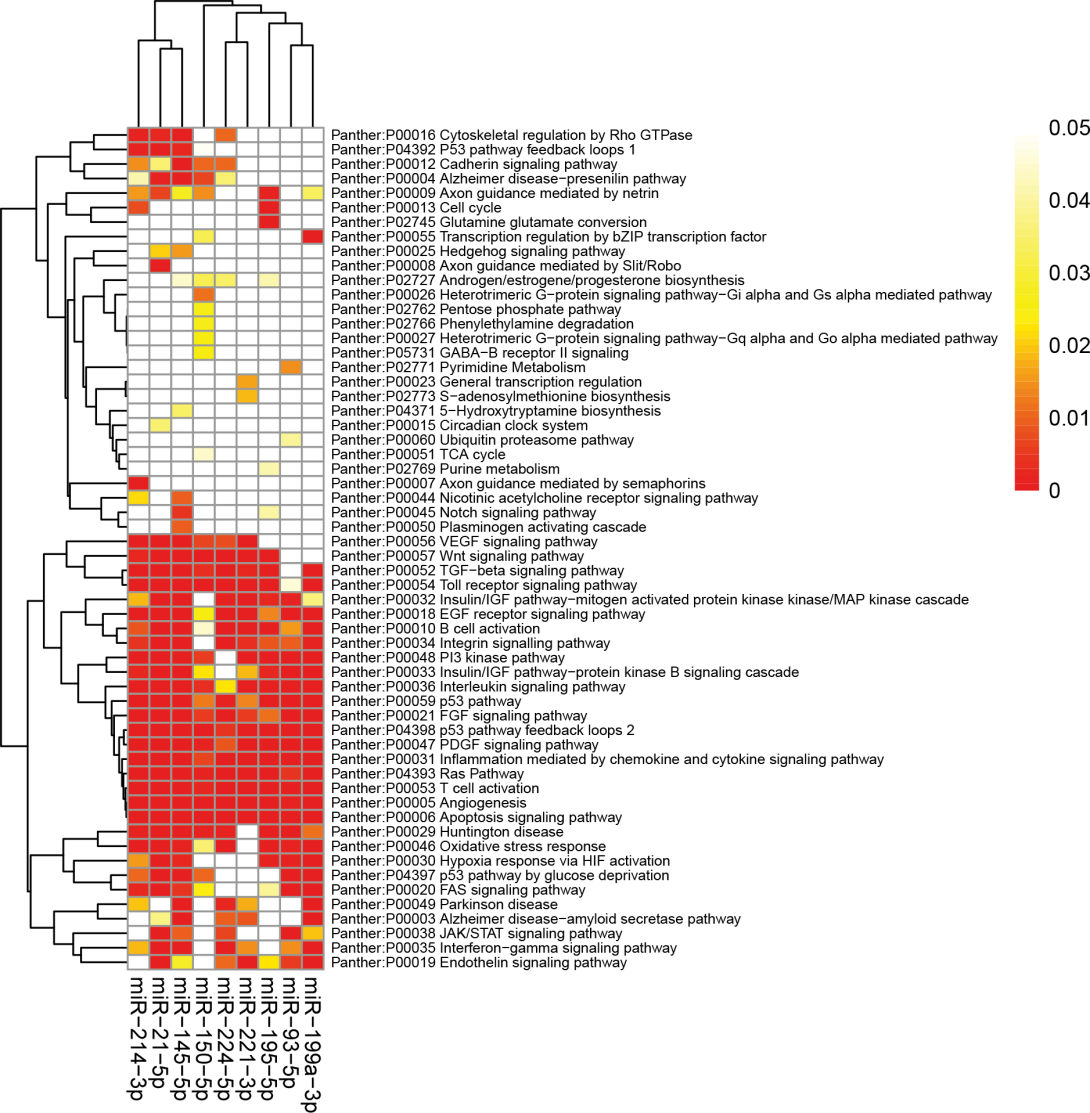
Panther pathway enrichment of targets by validated miRNAs The heatmap was constructed using the validated targets and GeneCodis web tool, which showed the results of panther pathway enrichment analysis. The intensity of color represents the FDR-corrected *p*-value. Clustering was performed using Pearson correlation and average linkage method. FDR, false discovery rate; miRNA, microRNA.

The top enriched panther pathways maps regulated by the miRNAs converge on apoptosis signaling pathway, interleukin signaling pathway, angiogenesis, PDGF signaling pathway, PI3 kinase pathway, p53 pathway feedback loops 2, and Ras Pathway, most of which are known to play an important role in carcinogentics (Figure 6). KEGG pathways that were significantly enriched with the miRNAs targets were mainly associated with cancer, e.g. pathways in cancer, chronic myeloid leukemia, pancreatic cancer, colorectal cancer, prostate cancer, and MAPK signaling pathway (Supplementary Figure 5). The miRNAs target genes are statistically enriched in GO processes of regulation of transcription. Ten pathways and GO processes most strongly enriched by integrated-signature miRNA targets were shown in Supplementary Table 2.

**Figure 7:**
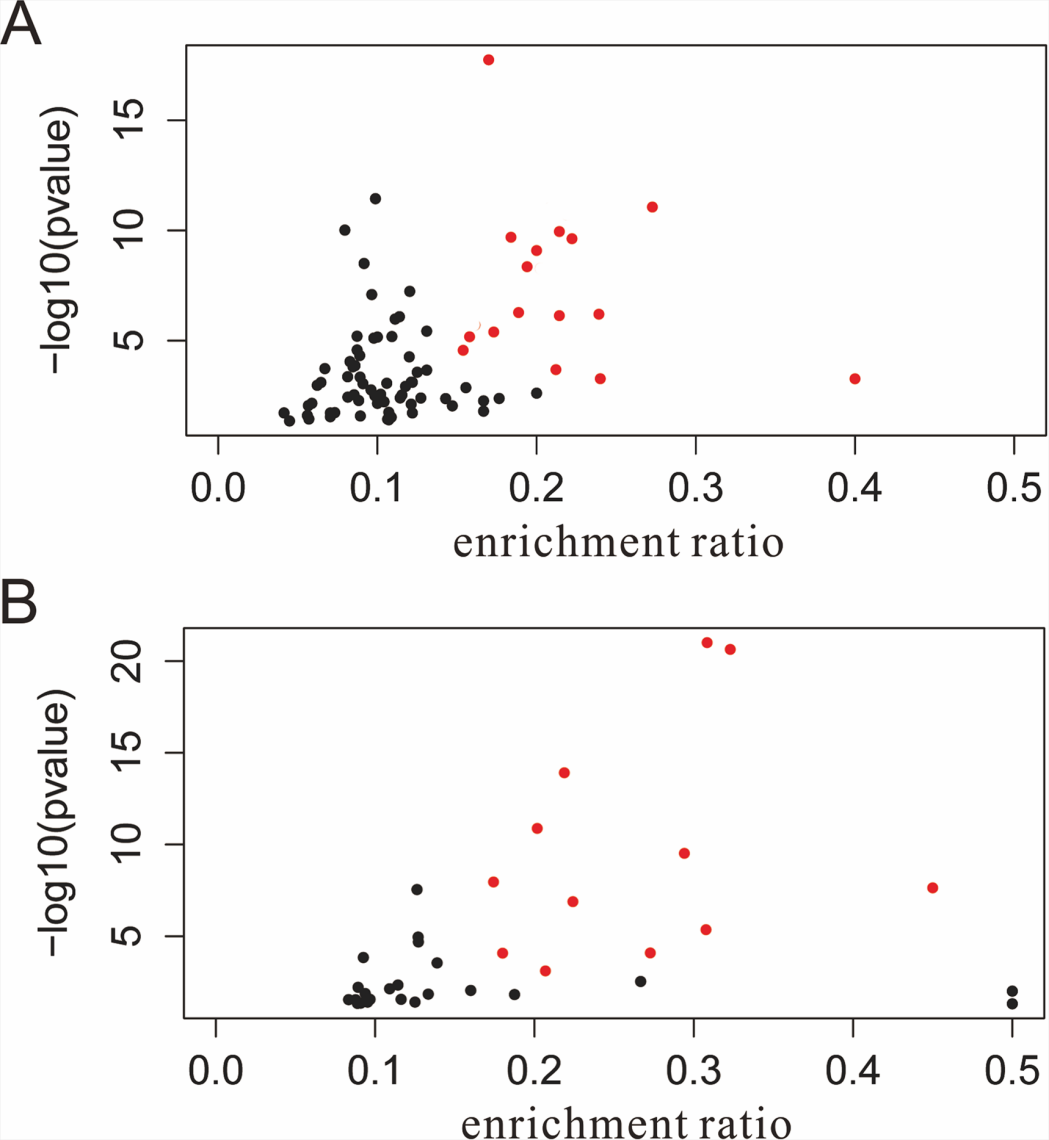
Volcano plot of pathways enriched with HCC-related genes **A.** KEGG pathways; **B.** Panther pathways. the red points indicate pathways of interest that display both large enrichment ratio (> 0.15, x-axis) as well as high statistical significance (*P* < 0.0001, y-axis). HCC, hepatocellular carcinoma; miRNA, microRNA.

Furthermore, to evaluate association between these pathways and HCC, the published papers which described HCC related constituent objects in the pathways were searched in PubMed. The pathway maps in which the constituent objects were supported in previous literature were considered to be HCC-related. After text mining, 39 Panther and 71 KEGG pathways pathways were found to be HCC-related. To visualize the most significantly enriched pathways, volcano plots were constructed by plotting the -log10 of *p*-value versus gene enrichment ratio on the y- and x-axes, respectively. Finally, 12 out of the 39 Panther pathways and 16 out of the 71 KEGG pathways were highly saturated with HCC (enrichment ratio > 0.15, *p*-value < 0.0001) (Figure 7, Table 2).

**Table 2:** The hepatocellular carcinoma highly saturated pathways by targets of validated microRNAs

Pathway	Enrichment Ratio	FDR
KEGG pathway		
04060: Cytokine-cytokine receptor interaction	0.17	1.80E–18
05215: Prostate cancer	0.20	3.46E–12
05220: Chronic myeloid leukemia	0.22	1.92E–11
05211: Renal cell carcinoma	0.21	1.12E–10
04012: ErbB signaling pathway	0.18	2.03E–10
05214: Glioma	0.22	2.37E–10
05212: Pancreatic cancer	0.20	8.14E–10
05218: Melanoma	0.20	8.14E–10
04115: p53 signaling pathway	0.19	4.42E–9
05223: Non-small cell lung cancer	0.19	5.32E–7
04930: Type II diabetes mellitus	0.24	6.31E–7
05219: Bladder cancer	0.21	7.35E–7
05210: Colorectal cancer	0.16	1.96E–6
04150: mTOR signaling pathway	0.17	4.05E–6
05221: Acute myeloid leukemia	0.16	6.67E–6
05213: Endometrial cancer	0.15	2.74E–5
Panther pathway		
P00002: Alpha adrenergic receptor signaling pathway	0.31	9.74E–22
P00032: Insulin/IGF pathway-mitogen activated protein kinase kinase/MAP kinase cascade	0.32	2.29E–21
P00023: General transcription regulation	0.22	1.22E–14
P00006: Apoptosis signaling pathway	0.20	1.32E–11
P00034: Integrin signalling pathway	0.29	3.02E–10
P00005: Angiogenesis	0.17	1.10E–8
P00038: JAK/STAT signaling pathway	0.45	2.30E–8
P00033: Insulin/IGF pathway-protein kinase B signaling cascade	0.22	1.30E–7
P00020: FAS signaling pathway	0.31	4.39E–6
P00025: Hedgehog signaling pathway	0.26	4.71E–6
P00045: Notch signaling pathway	0.27	8.13E–5
P00027: Heterotrimeric G-protein signaling pathway	0.18	8.38E–5

FDR, false discovery rate

## DISCUSSION

miRNA profiling efforts have often led to inconsistent results between the studies. Systematic review or meta-analysis has been done previously to determine differentially expressed genes in cancer at the gene level [32, 33]. However, such rigorous approach is often not possible due to the lack of cross-platform standardization of miRNA profiling technologies or the unavailability of raw data. In current study, we overcame the drawback of lack of agreement among miRNA expression profiling studies in HCC using RRA method which directly analyzed 26 prioritized miRNA lists detected from a total of 1250 paired HCC tissues and adjacent noncancerous tissues. The miRNAs would be re-ranked and their significance would be re-decided. A true combined *P* value were calculated for each miRNA. In addition, we determined the overlap among many studies using different platforms and observe which miRNAs are consistently reported as differentially expressed. Finally, an integrated-signature of 5 upregulated and 8 downregulated miRNAs was identified. The miRNAs from every dataset matched at least one of the integrated-signature miRNAs. These integrated-signiture miRNAs likely showed biological relevance to the tumorigenesis of HCC, as opposed to sporadically reported genes.

Futhermore, we attempted to divide the patients into smaller subgroups according to etilogies of HCC. Most cases of HCC were secondary to either a viral hepatitis infection (hepatitis B or C) or cirrhosis (alcoholism being the most common cause of hepatic cirrhosis). However, in the included datasets, 8 datasets derived from HBV related HCC samples, 1 from samples with HCV infection, while the others were muti-etiologies. Therefore, the only subset of datasets derived from HBV related HCC samples was reanalyzed. The results showed that 6 aberant miRNAs involving only HBV related HCC samples were included in the integrated-signature miRNAs with overall HCC datasets. This indicated that miRNA profile of the HBV related and non-HBV-related HCC might not be fundamentally different and the most significant aberrations probably reflect the mechanisms that are common to all subtypes of the disease.

Most integrated-signature miRNAs were known to be functionally associated with cancer development. miR-93 has been identified as a potential biomarker for detection of many cancers [34, 35]. Downregulation of miR-93 expression could reduce cell proliferation and clonogenicity of HepG2 cells. Furthermore, it is shown to directly target some tumor-suppressors [36, 37]. Many of validated targets of miR-93 were notably associated with the regulation of angiogenesis, apoptosis, and cell cycle regulation [38, 39]. Previous evidence has shown that miR-224 and miR-222 may function as an onco-miRNA in HCC cells by activating AKT signaling [15, 40]. Furthermore, miR-224, miR-222 and miR-221 were increased in HCC tissues and might be an independent poor prognostic factor [41, 42]. miR-21 has been validated as specific biomarker for many cancers, including HCC [43, 44]. miR-223, miR-199a, miR-145, miR-195 and miR-130a are commonly repressed in hepatocellular carcinoma. Downregulation of miR-214 contributes to HCC via activation of the HDGF paracrine pathway for tumor angiogenesis [8]. miR-214 could inhibit the tumorigenesis of HCC through suppression of β-catenin. miR-195 may exert its tumor suppressive function by decreasing the expression of multiple NF-κB downstream effectors by way of the direct targeting of IKKα and TAB3 in HCC. miR-150 is not extensively studied in HCC, but it functions as a tumour suppressor in human colorectal cancer by targeting c-Myb, an important pro-invasive molecule [45]. In addition, the results showed that corrected *p*-values of integrated-signature miRNAs were less than 1.0E-03 (Table 1). Interestingly, miR-122, as the most frequent miRNA in the liver, and a central player in liver biology and disease, was not part of HCC integrated- miRNA signature. miR-122 has been shown to be an essential regulator in the development of HCC [46]. miR-122 was found among downregulated miRNAs (4 studies), but did not reach the statistical significance in our integrated-analysis.

To determine whether these 13 miRNAs have been previously validated to have diagnostic/prognostic values as biomarkers in HCC, we also performed a validation experiment, and our data confirmed that miR-93-5p, miR-224-5p, miR-221-3p and miR-21-5p were up-regulated and miR-214-3p, miR-199a-3p, miR-195-5p, miR-150-5p and miR-145-5p were down-regulated in HCC tissues, which further supported the findings obtained in the present integrated bioinformatics analysis. Consistent with our initial analysis, 11 miRNAs were found to be significantly dysregulated in HCC tissues in TCGA data base, except miR-199a-5p and miR-199a-3p which were not listed. In current study, as was validated by qRT-PCR, the miRNAs were all expressed in liver tissues. Therefore, this miRNA panel might be novel potential biomarkers for the diagnosis of HCC. The miRNA based score using LASSO regression model provided a high classification accuracy of HCC tissue. Further studies could be performed to evaluate the diagnostic value of the miRNA expression signature in HCC. In addition, the target genes enrichment analysis suggested that the validated miRNAs were key regulatory drivers of the oncogenic process, which indicated very strong impact on several pathways related to signaling, regulation of transcription and tumor development. Therefore, these miRNAs may be good candidate biomarkers for diagnosing or monitoring remission during postoperative follow-up in HCC. In current study, tumor grades were also identified by some of the 13 miRNAs (miR-93-5p, miR-222-3p, miR-221-3p, miR-21-5p and miR-214-3p). Using LASSO regression, the signature can separate patients into well-differentiated and moderately/poorly differentiated tumor grades and may have clinical utility for decisions on patient management. However, none of the 13 most deregulated miRNAs was related with MVI in our initial analysis and TCGA data. Furthermore, we used Cox regression analysis to build a prognostic classifier, by which only miR-21 was selected.

The biological function of each validated miRNA were thoroughly investigated in our study. A single miRNA may target multiple target genes, and a specific mRNA may be regulated by many different miRNAs, which allow the miRNAs to induce changes in various pathways and processes and to present a further level of mechanism via which HCC may be induced. Supplementary Table 2 listed the ten most strongly enriched pathways and GO processes. The most significant pathways enriched in KEGG and Panther pathway by targets of rank aggregation miRNAs were pathways in cancer and apoptosis signaling pathway respectively, which highlighted the essential roles of miRNAs in cancer development. Regulation of transcription, known as the primary functions of miRNAs, was ranked first in the in the GO processes list. In functional enrichment analysis, when mapped to higher functional levels, inconsistent microRNA lists could fall within the same functional modules, pathways or networks and become more consistent. A better understanding of the functions of the miRNAs would advance their use in clinical settings. In addition to the known pathways in HCC tumorigenesis, we also performed text mining at pathway to evaluate the relevance of the enriched pathways in HCC. Sixteen KEGG pathways and 12 Panther pathways were shighly saturated with HCC (Figure 6).

Although our analysis was limited to comparison and validation between tumor and noncancerous tissue only, the 13 most significantly and consistently reported differentially expressed miRNAs could be used as potential diagnostic and/or prognostic biomarkers. In a clinical setting, sufficient sensitivity and specificity of the panel of miRNAs should be determined in the further well-designed clinical studies. Furthermore, targets prediction and functional enrichment analysis may provide a clue for elucidating the role of miRNAs in tumorigenesis of HCC and the precise underlying mechanisms. Taken together, the findings of the current study may have substantial clinical significance or implications.

In conclusion, a HCC associated microRNA expression signature, consisting of 11 highly significant and consistently dysregulated miRNAs, were identified in our integrated bioinformatics analysis and experimental validation study, which may be potential candidate biomarkers for HCC. The rigorous evaluation of integrated-miRNA signature and functional enrichment analysis of their targets were promising them as candidates for diagnostic markers of HCC. Further clinical and mechanistic studies focusing on these miRNAs are required for their clinical significance and the underlying mechanisms in tumorigenesis of HCC.

## MATERIALS AND METHODS

### Studies selection and datasets

Gene Expression Omnibus (GEO, www.ncbi.nlm.nih.gov/geo/), ISI Web of Science (thomsonreuters.com/web-of-science/), and ArrayExpress (www.ebi.ac.uk/arrayexpress) were searched for hepatocellular carcinoma miRNA expression profiling studies that had been published prior to December 31^st^, 2013. The search strategy was based on a combination of (mirna* OR microrna* OR mir-*) AND profil* AND ((liver AND (cancer* OR tumor* OR tumour* OR carcinoma)) OR (hepato* AND (cancer* OR tumor* OR tumour* OR carcinoma)). Citations of retrieved articles were also screened. Only original experimental articles published in English language were included. Full text of each study was carefully evaluated. The studies analyzed miRNA expression between HCC and noncancerous liver tissue in human were further analyzed. Expression studies of individual preselected candidate genes or studies using only cell lines were excluded. Studies that profiled different histologic subtypes but did not include noncancerous tissue were also excluded.

### Standardization of miRNA names

The lists of miRNAs with statistically significant (less than 0.05 was considered significant) expression changes between HCC and noncancerous liver tissue were extracted from the included studies. Authors were contacted for supplemental data, if the gene list was not available in the publication. For a comprehensive integrated analysis of miRNA expression, it is essential that the miRNA names are comparable across the studies and follow the same nomenclature. Because of the relative novelty of the miRNA profiling field and frequent updates in the miRBase, miRNA nomenclature can vary depending on when the study was conducted. Therefore, all miRNA names were standardized according to miRBase version 21 (http://www.mirbase.org/). Many traditional “major” miRNA names throughout the main text were redesignated according to miRBase database vesion 21. Viral miRNAs and non-miRNA probes were excluded from the analysis. Pre-miRNAs, reported in some of the studies, were used in the analyses after the standardization of precursor names.

### Datasets construction

The extracted miRNAs were ranked based on statistical test fold changes where reported, and *p*-values where old changes were not reported. The rank matrixes of upregulated and downregulated miRNAs lists were separate analyzed, which constructed the overall rank matrix. Furthermore, the rank of miRNA from the analysis of upregulated and downregulated miRNAs lists were both normalized, which was the original rank divided by the maximal possible rank in the study. In the normalized rank matrixes, *a* value was given to each miRNA, which was one minus normalized rank of miRNA from the analysis of upregulated gene lists, or normalized rank from analysis of downregulated gene lists. Value 0.5 means that this miRNA was not reported in that study, value above 0.5 means it is upregulated and value below 0.5 means that this miRNA is downregulated in that study.

### Statistical analysis

A novel RRA method implemented as an R package RobustRankAggreg was used to identify miRNAs that were ranked consistently better than expected by chance [47]. This method detects genes that are ranked consistently better than expected under null hypothesis of uncorrelated inputs and assigns a *P*-value for each gene. To assess the stability of acquired *p*-values, the leave one out cross-validation was applied on the robust rank aggregation algorithm. Analyses were repeated 10,000 times, and one random gene list was excluded from the analysis each time. Acquired *P*-values from each round for each miRNA were then averaged. All integrated-signature miRNAs that reached statistical significance after Bonferroni correction and were reported by at least 1/3 datasets were selected.

### Validation of the integrated-signature miRNAs using quantitative real-time PCR

To validate the results of integrated bioinformatics analysis, 11 pairs of fresh HCC and adjacent noncancerous liver tissues were obtained from 11 patients by experienced surgeons and examined by experienced pathologists at the the First Affiliated Hospital of Wenzhou Medical University between July and December, 2014. Written informed consent was obtained from all patients or their guardians. The samples were frozen immediately and stored in liquid nitrogen after being surgically resected. Clinical information was summarized in the Table 3. Total RNA was extracted using the Qiagen RNeasy Kit (QIAGEN GmbH, Germany) according to the manufacturer's instructions. First-strand complementary DNA (cDNA) was synthesized from 2 μl of total RNA using an oligo-dT primer and superscript II reverse transcriptase (Invitrogen). Then, quantification of the significantly up-regulated or down-regulated miRNAs was performed by real-time PCR, using SYBRRPremix Ex Taq TM (TakaRa). The primers of each dys-regulated miRNAs were listed in Supplementary Table 3. The primers for U6 were obtained from TakaRa. PCR was performed in a real-time PCR system (Applied Biosystems 7500) as follows: 95°C for 3 min followed by 35 cycles of 95°C for 5 sec, 60°C for 20 sec and 72°C for 30 sec and then 94°C for 1 min, 60°C for 1 min, with addition of a cycle for every 0.5°C. Expression values were normalized to those for U6 as a control. Relative fold changes of miRNA expression were calculated by the ΔΔCT method, and the values were expressed as 2^−ΔΔCT^. miRNAs with fold-change values ≥ 2 or ≤ 0.5 compared to adjacent noncancerous tissue were considered to be deregulated miRNAs in HCC.

**Table 3:** Characteristics of the patients

No.	gender	Age (year)	alpha-fetal protein	etiology	Cirrhosis	Child-Pugh class	Tumor size (cm)	Tumor grade	MVI
1	female	63	12.28	HBV	Present	A	3	G1	Absent
2	male	56	593.62	HBV	Present	A	3.5	G3	Absent
3	male	63	10.04	HBV/Alcohol	Absent	A	2	G1	Absent
4	male	48	1.69	HBV	Present	B	7	G1	Absent
5	male	50	6.34	HBV/Alcohol	Absent	B	4	G4	Present
6	male	42	1024.96	HBV	Present	A	3	G3	Present
7	female	64	87.05	HBV	Present	A	2	G3	Absent
8	male	55	450.72	HBV/Alcohol	Present	B	12	G3	Present
9	male	65	2.26	HBV	Present	A	13	G2	Present
10	male	49	22663.00	HBV/Alcohol	Present	A	9	G2	Present
11	female	71	3.66	HBV	Present	A	4	G1	Absent

HBV, hepatitis B virus; MVI, microscopic vascular invasion

The paired test was used to examine the difference of miRNA expression levels between tumor and adjacent nontumor liver tissues. The prognosis of HCC strongly depends upon nuclear grade and the presence of microscopic vascular invasion (MVI). Therefore, the difference of miRNA expression levels were also tested in samples with or without MVI, different tumor grades and survival. MVI was defined by the presence of tumour emboli within either the central hepatic vein, the portal, or the large capsular vessels [48]. Edmondson and Steiner's nuclear grades were used to classify the tumor grade, in which grades 1 and 2 were defined as well-differentiated, and grades 3 and 4 as moderately/poorly differentiated [49]. The results were validated on the TCGA datasets. miRNA expression data and corresponding clinical information for HCC dataset were downloaded from TCGA data portal in January 2015. TCGA data are classified by data type (clinical, mutations, gene expression) and data level, to allow structured access to this resource with appropriate patient privacy protection (Supplementary Table 4). This study meets the publication guidelines provided by TCGA. The miRNA expression profiling was performed using the Illumina HiSeq 2000 miRNA sequencing platforms (Illumina Inc, San Diego, CA). The miRNA expression level was demonstrated as reads per million miRNA mapped data. The miRNA expression analyses were performed using BRB-ArrayTools (version 4.4) developed by Dr. Richard Simon and the BRB-ArrayTools Development Team. In brief, the miRNAs with missing data exceeded 10% of all subjects were excluded from the dataset and the expression level of each individual miRNA was log2-transformed for further analysis. The predicted performances of the validated miRNAs for classifying HCC, MVI, and tumour grade were estimated on the TCGA datasets using ROC curve. The TCGA samples were assessed using a LASSO penalized regression analysis to predict HCC, MVI, tumor grade and survival using microRNA expression with leave-one-out cross-validation using R software (v3.1.2) and the Lars package (v1.2) [50]. A risk score was generated using the sum of microRNA expression values weighted by the coefficients from the LASSO regression, as described. The statistical analyses were performed using the SPSS 18.0 (SPSS Inc.). Statistical significance was defined as *p* < 0.05.

For survival analysis, we used the Kaplan-Meier method to analysis the correlation between overall survival and the miRNAs, and the logrank test was used to compare survival curves. The optimum cut-off value for the miRNAs using X-tile plots based on the association with mortality of the patients. X-tile plots provide a single and intuitive method to assess the association between variables and survival. The X-tile program can automatically select the optimum data cut point according to the highest χ^2^ value (minimum *p* value) defined by Kaplan-Meier survival analysis and log-rank test [51]. We did the X-tile plots using the X-tile software version 3.6.1 (Yale University School of Medicine, New Haven, CT, USA).

### miRNA target prediction

The putative targets of integrated-signature miRNAs were predicted using databases utilizing three different target prediction algorithms: TargetScan v6.2 (http://www.targetscan.org/), RNA22 (https://cm.jefferson.edu/rna22v2/), miRDB (http://www.mirdb.org/miRDB/), RNAhybrid (http://bibiserv.techfak.uni-bielefeld.de/rnahybrid/) and DIANA-microT-CDS Web Server v5.0 (http://diana.imis.athena-innovation.gr/DianaTools/index.php?r=microT_CDS/index). DIANA algorithm predictions were performed using miTG score threshold 0.7 (strict setting). Only genes with target sites in 3′UTR were used. Validated targets were acquired from TarBase v6.0 database (http://diana.imis.athena-innovation.gr/DianaTools/index.php?r=tarbase/index) and miRwalk (http://www.umm.uni-heidelberg.de/apps/zmf/mirwalk/mirnatargetpub.html). Consensus targets were then defined as genes predicted by at least 4 algorithms plus validated targets from TarBase and starBase.

### Enrichment analysis

Enrichment analyses for Panther and KEGG pathways and Gene Ontology terms were carried out with GeneCodis web tool (http://genecodis.dacya.ucm.es/) [52]. Predicted target genes for each miRNA were used as input and false discovery rate (FDR)-corrected *p*-values were visualized as a heatmap. Clustering of the heatmap was based on Pearson correlation and average linkage. Furthermore, the association between the pathways affected by altered expression of miRNAs and HCC was evaluated.

## SUPPLEMENTARY FIGURES AND TABLES



## List of abbreviations

FDR: false discovery rate
HBV: hepatitis B virus
HCC: hepatocellular carcinoma
HCV: hepatitis C virus
miRNA: hazard ratio
HR: microRNA
MVI: microscopic vascular invasion
ROC: receiver operating characteristic
RRA: robust rank aggregation
TCGA: Tumor Cancer Genome Atlas

## References

[1] Kansagara D, Papak J, Pasha AS, O'Neil M, Freeman M, Relevo R, Quinones A, Motu'apuaka M, Jou JH (2014). Screening for hepatocellular carcinoma in chronic liver disease: a systematic review. Annals of internal medicine.

[2] Song JL, Nigam P, Tektas SS, Selva E (2015). microRNA regulation of Wnt Signaling Pathways in Development and Disease. Cell Signal.

[3] Murakami Y, Yasuda T, Saigo K, Urashima T, Toyoda H, Okanoue T, Shimotohno K (2006). Comprehensive analysis of microRNA expression patterns in hepatocellular carcinoma and non-tumorous tissues. Oncogene.

[4] Li W, Xie L, He X, Li J, Tu K, Wei L, Wu J, Guo Y, Ma X, Zhang P, Pan Z, Hu X, Zhao Y, Xie H, Jiang G, Chen T (2008). Diagnostic and prognostic implications of microRNAs in human hepatocellular carcinoma. Int J Cancer.

[5] Huang YS, Dai Y, Yu XF, Bao SY, Yin YB, Tang M, Hu CX (2008). Microarray analysis of microRNA expression in hepatocellular carcinoma and non-tumorous tissues without viral hepatitis. J Gastroenterol Hepatol.

[6] Budhu A, Jia HL, Forgues M, Liu CG, Goldstein D, Lam A, Zanetti KA, Ye QH, Qin LX, Croce CM, Tang ZY, Wang XW (2008). Identification of metastasis-related microRNAs in hepatocellular carcinoma. Hepatology.

[7] Li S, Fu H, Wang Y, Tie Y, Xing R, Zhu J, Sun Z, Wei L, Zheng X (2009). MicroRNA-101 regulates expression of the v-fos FBJ murine osteosarcoma viral oncogene homolog (FOS) oncogene in human hepatocellular carcinoma. Hepatology.

[8] Shih TC, Tien YJ, Wen CJ, Yeh TS, Yu MC, Huang CH, Lee YS, Yen TC, Hsieh SY (2012). MicroRNA-214 downregulation contributes to tumor angiogenesis by inducing secretion of the hepatoma-derived growth factor in human hepatoma. J Hepatol.

[9] Wong QW, Lung RW, Law PT, Lai PB, Chan KY, To KF, Wong N (2008). MicroRNA-223 is commonly repressed in hepatocellular carcinoma and potentiates expression of Stathmin1. Gastroenterology.

[10] Yang L, Ma Z, Wang D, Zhao W, Chen L, Wang G (2010). MicroRNA-602 regulating tumor suppressive gene RASSF1A is overexpressed in hepatitis B virus-infected liver and hepatocellular carcinoma. Cancer Biol Ther.

[11] Vosa U, Kolde R, Vilo J, Metspalu A, Annilo T (2014). Comprehensive meta-analysis of microRNA expression using a robust rank aggregation approach. Methods in molecular biology.

[12] Wong CM, Wong CC, Lee JM, Fan DN, Au SL, Ng IO (2012). Sequential alterations of microRNA expression in hepatocellular carcinoma development and venous metastasis. Hepatology.

[13] Gramantieri L, Ferracin M, Fornari F, Veronese A, Sabbioni S, Liu CG, Calin GA, Giovannini C, Ferrazzi E, Grazi GL, Croce CM, Bolondi L, Negrini M (2007). Cyclin G1 is a target of miR-122a, a microRNA frequently down-regulated in human hepatocellular carcinoma. Cancer Res.

[14] Connolly E, Melegari M, Landgraf P, Tchaikovskaya T, Tennant BC, Slagle BL, Rogler LE, Zavolan M, Tuschl T, Rogler CE (2008). Elevated expression of the miR-17–92 polycistron and miR-21 in hepadnavirus-associated hepatocellular carcinoma contributes to the malignant phenotype. Am J Pathol.

[15] Wong QW, Ching AK, Chan AW, Choy KW, To KF, Lai PB, Wong N (2010). MiR-222 overexpression confers cell migratory advantages in hepatocellular carcinoma through enhancing AKT signaling. Clin Cancer Res.

[16] Jiang R, Deng L, Zhao L, Li X, Zhang F, Xia Y, Gao Y, Wang X, Sun B (2011). miR-22 promotes HBV-related hepatocellular carcinoma development in males. Clin Cancer Res.

[17] Wang W, Zhao LJ, Tan YX, Ren H, Qi ZT (2012). Identification of deregulated miRNAs and their targets in hepatitis B virus-associated hepatocellular carcinoma. World J Gastroenterol.

[18] Wei R, Huang GL, Zhang MY, Li BK, Zhang HZ, Shi M, Chen XQ, Huang L, Zhou QM, Jia WH, Zheng XF, Yuan YF, Wang HY (2013). Clinical significance and prognostic value of microRNA expression signatures in hepatocellular carcinoma. Clin Cancer Res.

[19] Mizuguchi Y, Mishima T, Yokomuro S, Arima Y, Kawahigashi Y, Shigehara K, Kanda T, Yoshida H, Uchida E, Tajiri T, Takizawa T (2011). Sequencing and bioinformatics-based analyses of the microRNA transcriptome in hepatitis B-related hepatocellular carcinoma. PLoS One.

[20] Burchard J, Zhang C, Liu AM, Poon RT, Lee NP, Wong KF, Sham PC, Lam BY, Ferguson MD, Tokiwa G, Smith R, Leeson B, Beard R, Lamb JR, Lim L, Mao M (2010). microRNA-122 as a regulator of mitochondrial metabolic gene network in hepatocellular carcinoma. Mol Syst Biol.

[21] Diaz G, Melis M, Tice A, Kleiner DE, Mishra L, Zamboni F, Farci P (2013). Identification of microRNAs specifically expressed in hepatitis C virus-associated hepatocellular carcinoma. Int J Cancer.

[22] Jiang J, Gusev Y, Aderca I, Mettler TA, Nagorney DM, Brackett DJ, Roberts LR, Schmittgen TD (2008). Association of MicroRNA expression in hepatocellular carcinomas with hepatitis infection, cirrhosis, and patient survival. Clin Cancer Res.

[23] Wang Y, Lee AT, Ma JZ, Wang J, Ren J, Yang Y, Tantoso E, Li KB, Ooi LL, Tan P, Lee CG (2008). Profiling microRNA expression in hepatocellular carcinoma reveals microRNA-224 up-regulation and apoptosis inhibitor-5 as a microRNA-224-specific target. J Biol Chem.

[24] Huang XH, Wang Q, Chen JS, Fu XH, Chen XL, Chen LZ, Li W, Bi J, Zhang LJ, Fu Q, Zeng WT, Cao LQ, Tan HX, Su Q (2009). Bead-based microarray analysis of microRNA expression in hepatocellular carcinoma: miR-338 is downregulated. Hepatol Res.

[25] Liu WH, Yeh SH, Lu CC, Yu SL, Chen HY, Lin CY, Chen DS, Chen PJ (2009). MicroRNA-18a prevents estrogen receptor-alpha expression, promoting proliferation of hepatocellular carcinoma cells. Gastroenterology.

[26] Chung GE, Yoon JH, Myung SJ, Lee JH, Lee SH, Lee SM, Kim SJ, Hwang SY, Lee HS, Kim CY (2010). High expression of microRNA-15b predicts a low risk of tumor recurrence following curative resection of hepatocellular carcinoma. Oncol Rep.

[27] Borel F, Han R, Visser A, Petry H, van Deventer SJ, Jansen PL, Konstantinova P (2012). Adenosine triphosphate-binding cassette transporter genes up-regulation in untreated hepatocellular carcinoma is mediated by cellular microRNAs. Hepatology.

[28] Huang Y, Chen HC, Chiang CW, Yeh CT, Chen SJ, Chou CK (2012). Identification of a two-layer regulatory network of proliferation-related microRNAs in hepatoma cells. Nucleic Acids Res.

[29] Sato F, Hatano E, Kitamura K, Myomoto A, Fujiwara T, Takizawa S, Tsuchiya S, Tsujimoto G, Uemoto S, Shimizu K (2011). MicroRNA profile predicts recurrence after resection in patients with hepatocellular carcinoma within the Milan Criteria. PLoS One.

[30] Alencar AJ, Malumbres R, Kozloski GA, Advani R, Talreja N, Chinichian S, Briones J, Natkunam Y, Sehn LH, Gascoyne RD, Tibshirani R, Lossos IS (2011). MicroRNAs are independent predictors of outcome in diffuse large B-cell lymphoma patients treated with R-CHOP. Clin Cancer Res.

[31] Waldron L, Pintilie M, Tsao MS, Shepherd FA, Huttenhower C, Jurisica I (2011). Optimized application of penalized regression methods to diverse genomic data. Bioinformatics.

[32] Griffith OL, Melck A, Jones SJ, Wiseman SM (2006). Meta-analysis and meta-review of thyroid cancer gene expression profiling studies identifies important diagnostic biomarkers. Journal of clinical oncology : official journal of the American Society of Clinical Oncology.

[33] Chan SK, Griffith OL, Tai IT, Jones SJ (2008). Meta-analysis of colorectal cancer gene expression profiling studies identifies consistently reported candidate biomarkers. Cancer epidemiology, biomarkers & prevention : a publication of the American Association for Cancer Research, cosponsored by the American Society of Preventive Oncology.

[34] Zhu W, He J, Chen D, Zhang B, Xu L, Ma H, Liu X, Zhang Y, Le H (2014). Expression of miR-29c, miR-93, and miR-429 as potential biomarkers for detection of early stage non-small lung cancer. PLoS One.

[35] Wang S, Xiang J, Li Z, Lu S, Hu J, Gao X, Yu L, Wang L, Wang J, Wu Y, Chen Z, Zhu H (2015). A plasma microRNA panel for early detection of colorectal cancer. Int J Cancer.

[36] Singh B, Ronghe AM, Chatterjee A, Bhat NK, Bhat HK (2013). MicroRNA-93 regulates NRF2 expression and is associated with breast carcinogenesis. Carcinogenesis.

[37] Du L, Zhao Z, Ma X, Hsiao TH, Chen Y, Young E, Suraokar M, Wistuba I, Minna JD, Pertsemlidis A (2014). miR-93-directed downregulation of DAB2 defines a novel oncogenic pathway in lung cancer. Oncogene.

[38] Kim YK, Yu J, Han TS, Park SY, Namkoong B, Kim DH, Hur K, Yoo MW, Lee HJ, Yang HK, Kim VN (2009). Functional links between clustered microRNAs: suppression of cell-cycle inhibitors by microRNA clusters in gastric cancer. Nucleic Acids Res.

[39] Fang L, Du WW, Yang W, Rutnam ZJ, Peng C, Li H, O'Malley YQ, Askeland RW, Sugg S, Liu M, Mehta T, Deng Z, Yang BB (2012). MiR-93 enhances angiogenesis and metastasis by targeting LATS2. Cell Cycle.

[40] Ma D, Tao X, Gao F, Fan C, Wu D (2012). miR-224 functions as an onco-miRNA in hepatocellular carcinoma cells by activating AKT signaling. Oncol Lett.

[41] Yu L, Zhang J, Guo X, Li Z, Zhang P (2014). MicroRNA-224 upregulation and AKT activation synergistically predict poor prognosis in patients with hepatocellular carcinoma. Cancer epidemiology.

[42] Rong M, Chen G, Dang Y (2013). Increased miR-221 expression in hepatocellular carcinoma tissues and its role in enhancing cell growth and inhibiting apoptosis *in vitro*. BMC Cancer.

[43] Wang LJ, He CC, Sui X, Cai MJ, Zhou CY, Ma JL, Wu L, Wang H, Han SX, Zhu Q (2015). MiR-21 promotes intrahepatic cholangiocarcinoma proliferation and growth *in vitro* and *in vivo* by targeting PTPN14 and PTEN. Oncotarget.

[44] Zhang K, Chen J, Chen D, Huang J, Feng B, Han S, Chen Y, Song H, De W, Zhu Z, Wang R, Chen L (2014). Aurora-A promotes chemoresistance in hepatocelluar carcinoma by targeting NF-kappaB/microRNA-21/PTEN signaling pathway. Oncotarget.

[45] Feng J, Yang Y, Zhang P, Wang F, Ma Y, Qin H, Wang Y (2014). miR-150 functions as a tumour suppressor in human colorectal cancer by targeting c-Myb. Journal of cellular and molecular medicine.

[46] Nakao K, Miyaaki H, Ichikawa T (2014). Antitumor function of microRNA-122 against hepatocellular carcinoma. Journal of gastroenterology.

[47] Kolde R, Laur S, Adler P, Vilo J (2012). Robust rank aggregation for gene list integration and meta-analysis. Bioinformatics.

[48] Vauthey JN, Lauwers GY, Esnaola NF, Do KA, Belghiti J, Mirza N, Curley SA, Ellis LM, Regimbeau JM, Rashid A, Cleary KR, Nagorney DM (2002). Simplified staging for hepatocellular carcinoma. Journal of clinical oncology : official journal of the American Society of Clinical Oncology.

[49] Nzeako UC, Goodman ZD, Ishak KG (1995). Comparison of tumor pathology with duration of survival of North American patients with hepatocellular carcinoma. Cancer.

[50] Usai MG, Goddard ME, Hayes BJ (2009). LASSO with cross-validation for genomic selection. Genetics research.

[51] Camp RL, Dolled-Filhart M, Rimm DL (2004). X-tile: a new bio-informatics tool for biomarker assessment and outcome-based cut-point optimization. Clin Cancer Res.

[52] Nogales-Cadenas R, Carmona-Saez P, Vazquez M, Vicente C, Yang X, Tirado F, Carazo JM, Pascual-Montano A (2009). GeneCodis: interpreting gene lists through enrichment analysis and integration of diverse biological information. Nucleic Acids Res.

