# Supplementary material for: Hepatocellular carcinoma associated microRNA expression signature: integrated bioinformatics analysis, experimental validation and clinical significance
Source: Oncotarget. 2015 Jun 30;6(28):25093–108. doi: 10.18632/oncotarget.4437 (PMC4694817; doi:10.18632/oncotarget.4437)
Supplement: Supplementary file 1 [file oncotarget-06-25093-s001.pdf]

SUPPLEMENTARY FIGURES AND TABLES

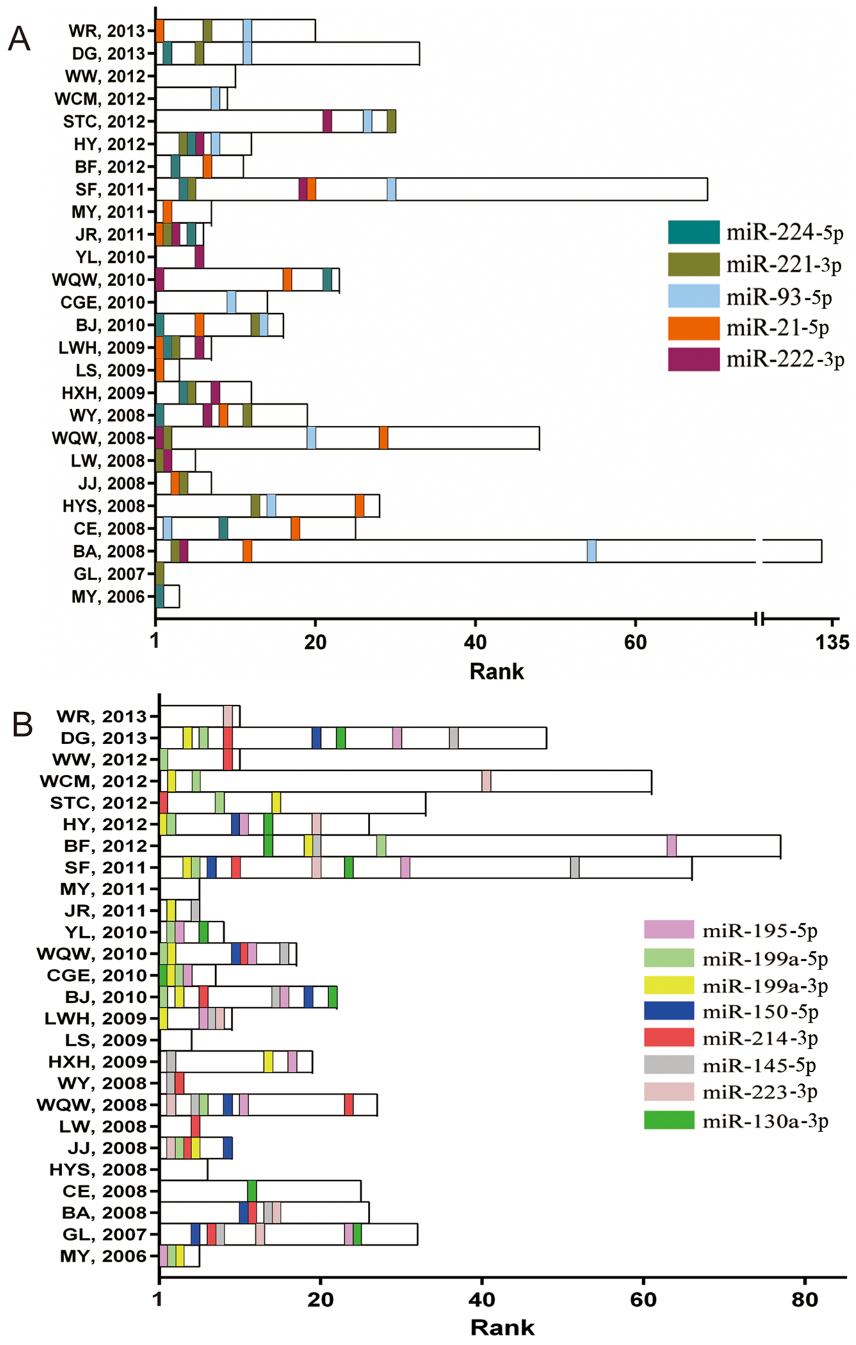

Supplementary Figure 1: Positions of HCC integrated-signature miRNAs identified by robust rank aggregation analysis. The number of significantly upregulated **A.** or downregulated **B.** miRNAs was designated in the boxes. The rank scale was shown on the left. HCC, hepatocellular carcinoma; miRNA, microRNA.

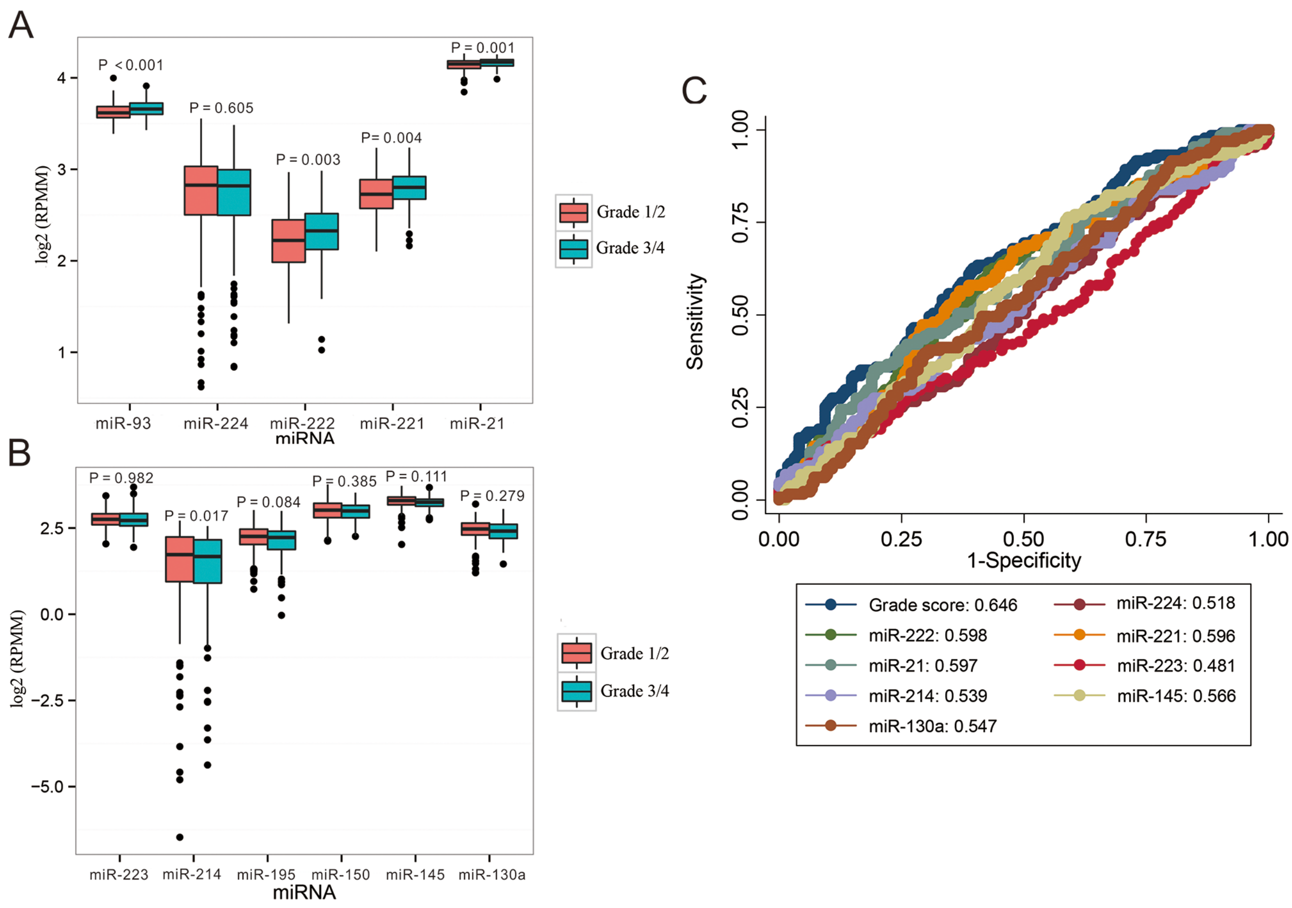

Supplementary Figure 2: miRNAs expression in different tumor grades on the TCGA dataset. A. Upregulated miRNAs expression. **B.** Downregulated miRNAs expression. **C.** Performances of the 5 miRNAs in tumor grade classification. For boxplots, expression values of miRNAs were log2-transformed and box width was proportional to the square root of sample size in each variant. Grade score was built using LASSO regression model by R software: 0.0427E_miR-222 + 0.0030E_miR-221 + 0.0763E_miR-21 - 0.0184E_miR-214-3p + 0.0098E_miR-130a. E_miR-n = Log 2 (expression of microRNA n). Grade 1/2: well-differentiated tumor grade, Grade 3/4: moderately/poorly differentiated tumor grade. miRNA, microRNA; TCGA, Tumor Cancer Genome Atlas.

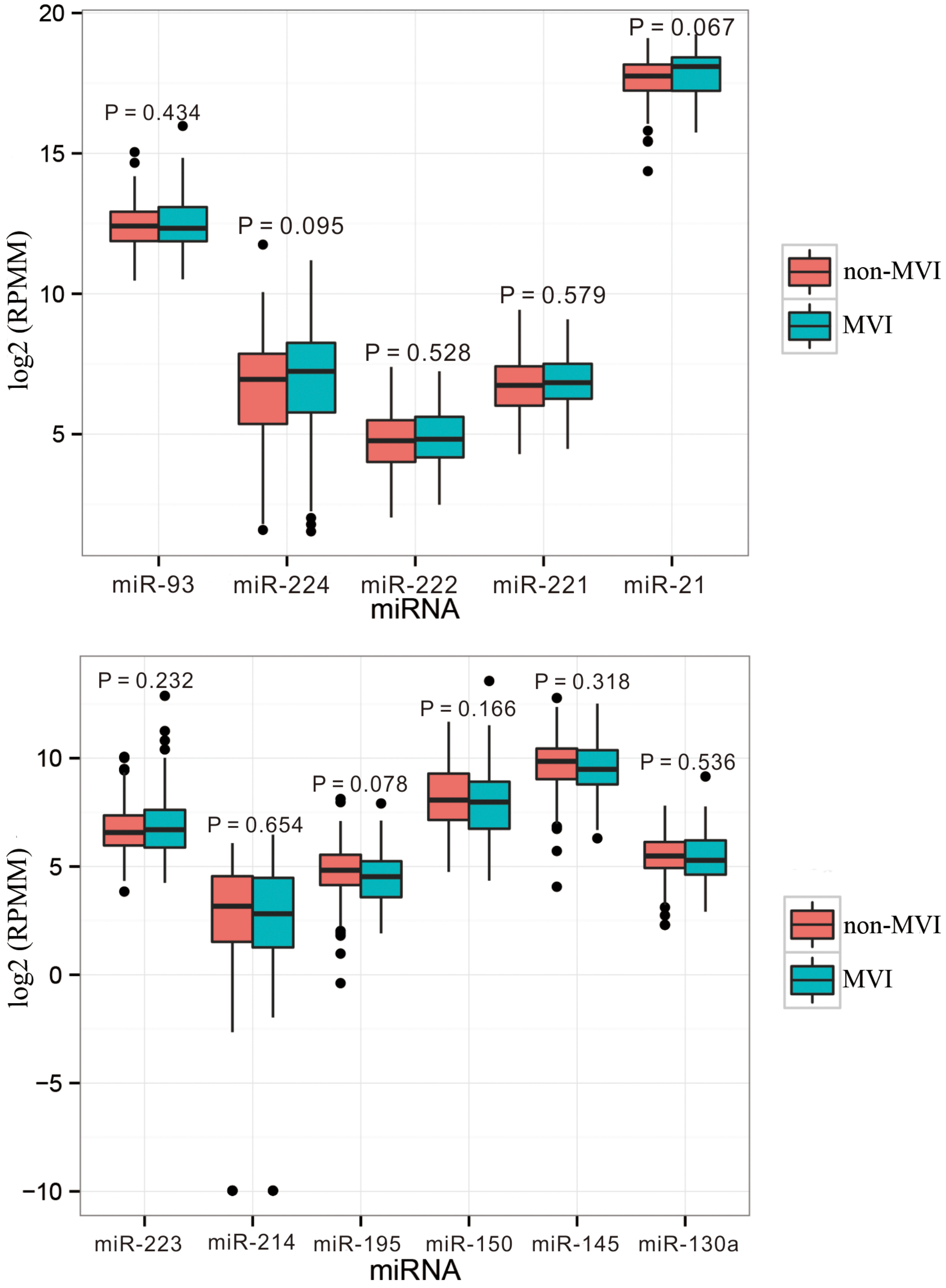

Supplementary Figure 3: miRNAs expression in presence and absence of microscopic vascular invasion on the TCGA dataset. A. upregulated miRNAs expression on the TCGA dataset. **B.** downregulated miRNAs expression on the TCGA dataset. For boxplots, expression values of miRNAs were log2-transformed and box width was proportional to the square root of sample size in each variant. MVI, microscopic vascular invasion. miRNA, microRNA; TCGA, Tumor Cancer Genome Atlas.

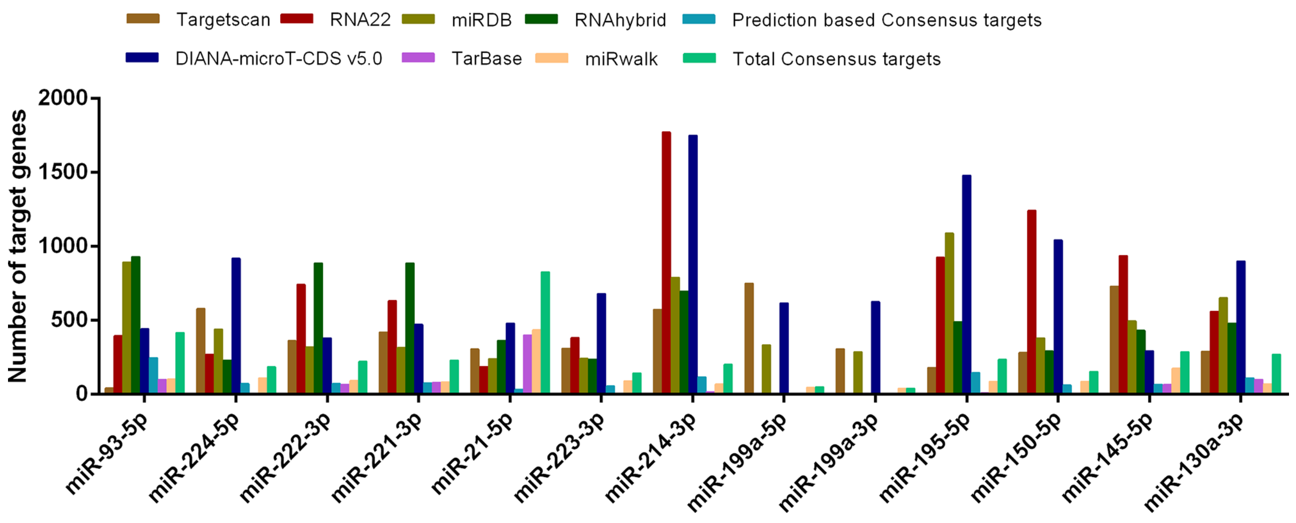

Supplementary Figure 4: The distribution of target counts predicted by target prediction algorithms. Prediction based consensus targets were defined as genes predicted by at least 4 algorithms. Consensus targets were defined as genes predicted by at least 4 algorithms plus validated targets from TarBase and starBase.

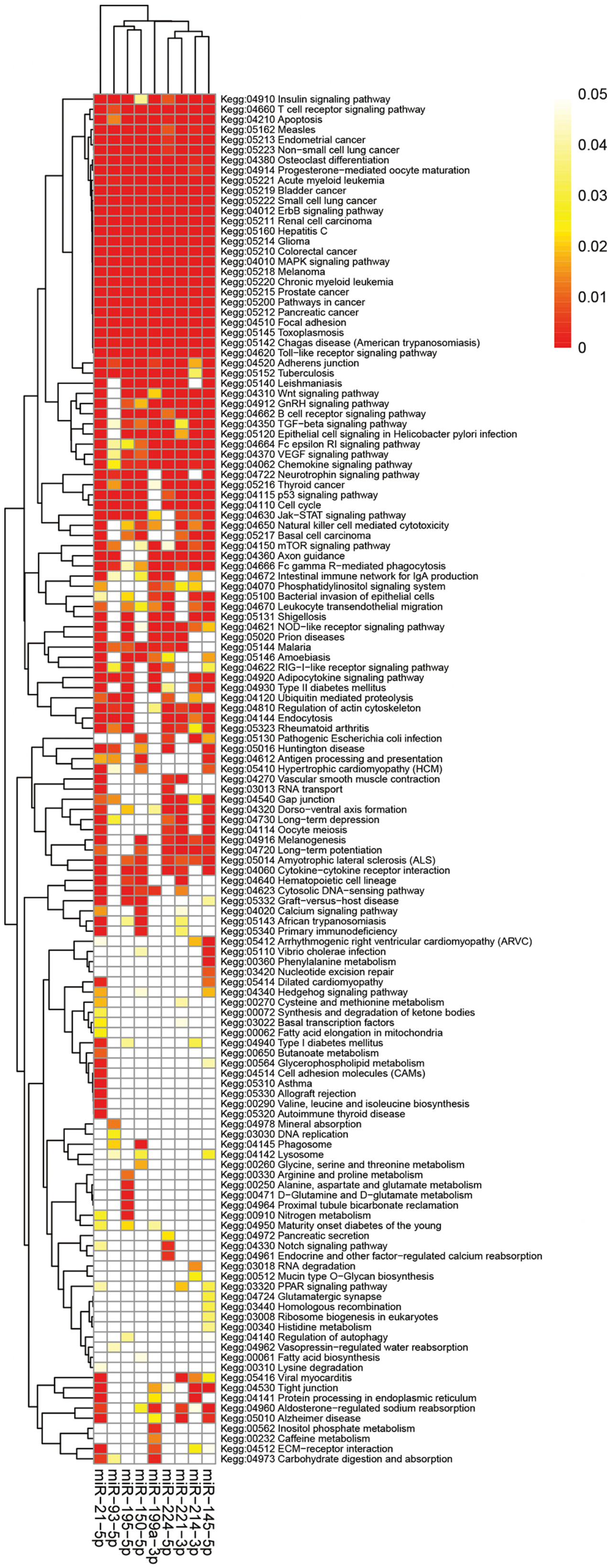

Supplementary Figure 5: Kegg pathway enrichment of targets by validated miRNAs. The heatmap was constructed using the validated targets and GeneCodis web tool, which showed the results of panther pathway enrichment analysis. The intensity of color represents the FDR-corrected *p*-value. Clustering was performed using Pearson correlation and average linkage method. FDR, false discovery rate; miRNA, microRNA.

Supplementary Table 1: Characteristics of included datasets

First author, year	Ref.	Region	Assay type	Number of miRNA probes	Number of samples	Etiology
MY, 2006	3	Asia	Custom microarray	386	25 pairs	mixed
GL, 2007	13	Europe	Custom microarray	381	13 pairs	mixed
BA, 2008	6	North America	Custom microarray	482	241 pairs	mixed
CE, 2008	14	North America	miRCURY LNA microRNA Array	278	19 pairs	HBV
HYS, 2008	5	Asia	Custom microarray	331	10 pairs	mixed
JJ, 2008	22	North America	System Biosciences, Taqman	182	54 pairs	mixed
LW, 2008	4	Asia	Custom microarray	509	78 pairs	mixed
WQW, 2008	9	Asia	mirMAX array	182	42 pairs	mixed
WY, 2008	23	Asia	System Biosciences, Taqman	157	19 pairs	mixed
HXH, 2009	24	Asia	mirMASA technique	114	20 pairs	mixed
LS, 2009	7	Asia	QuantiTect PCR	453	20 pairs	mixed
LWH, 2009	25	Asia	System Biosciences, Taqman	233	80 pairs	mixed
BJ, 2010	20	North America	Affymetrix GeneChip array	220	96 pairs	HBV
CGE, 2010	26	Asia	Custom microarray	449	25 pairs	mixed
WQW, 2010	15	Asia	System Biosciences, Taqman	156	12 pairs	HBV
YL, 2010	10	Asia	Exiqon miRCURY LNA array	730	8 pairs	HBV
JR, 2011	16	Asia	SOLiD sequencing	182	116 pairs	HBV
MY, 2011	19	Asia	GS 454 technology	208818	22 pairs	mixed
SF, 2011	29	Asia	Exiqon miRCURY LNA array	857	73 pairs	mixed
BF, 2012	27	Europe	System Biosciences, Taqman	378	19 pairs	mixed
HY, 2012	28	Asia	Affymetrix GeneChip array	270	20 pairs	mixed
STC, 2012	8	Asia	Illumina microarray V2	1145	81 pairs	mixed
WCM, 2012	12	Asia	qPCR, Mega Plex kit	664	20 pairs	mixed
WW, 2012	17	Asia	qPCR, Mega Plex kit	667	18 pairs	HBV
DG, 2013	21	Europe	Affymetrix GeneChip array	2226	9 pairs	HCV
WR, 2013	18	Asia	Custom microarray	683	110 pairs	HBV

HBV, hepatitis B virus; HCV, hepatitis C virus; miRNA, microRNA

Supplementary Table 2: The most significant 10 pathways and GO processes enriched by targets of rank aggregation microRNAs

Panther pathways	FDR	Targets
P00006: Apoptosis signaling pathway	9.745E–22	33
P00036: Interleukin signaling pathway	2.29E–21	31
P00005: Angiogenesis	7.88E–19	35
P00047: PDGF signaling pathway	1.22E–14	28
P00048: PI3 kinase pathway	2.03E–13	15
P04398: p53 pathway feedback loops 2	5.08E–13	17
P00059: p53 pathway	5.15E–13	21
P00052: TGF-beta signaling pathway	1.03E–12	22
P04393: Ras Pathway	3.45E–12	19
P00018: EGF receptor signaling pathway	1.32E–11	23
KEGG pathways		
05200: Pathways in cancer	6.70E–56	90
05220: Chronic myeloid leukemia	6.065E–31	35
05212: Pancreatic cancer	1.83E–30	34
05210: Colorectal cancer	2.30E–28	31
05215: Prostate cancer	3.07E–25	33
05162: Measles	1.12E–22	29
05142: Chagas disease	8.14E–22	32
04510: Focal adhesion	3.87E–20	27
05222: Small cell lung cancer	5.91E–20	28
04010: MAPK signaling pathway	5.35E–19	45
GO processes		
GO: 0045944: positive regulation of transcription from RNA polymerase II promoter	2.74E–71	133
GO: 0045893: positive regulation of transcription	3.54E–43	93
GO: 0043066: negative regulation of apoptotic process	9.15E–35	65
GO: 0000122: negative regulation of transcription from RNA polymerase II promoter	6.33E–30	73
GO: 0045892: negative regulation of transcription	2.10E–29	71
GO: 0008284: positive regulation of cell proliferation	2.75E–27	65
GO: 0006355: regulation of transcription	2.81E–27	145
GO: 0042493: response to drug	4.99E–25	57
GO: 0008285: negative regulation of cell proliferation	7.55E–24	59
GO: 0043065: positive regulation of apoptotic process	3.82E–20	40

FDR, false discovery rate

Supplementary Table 3: The primers of each miRNAs from QIAGEN

miRNAs	Product code
hsa-miR-93-5p	MS00003346
hsa-miR-224-5p	MS00003878
hsa-miR-222-3p	MS00007609
hsa-miR-221-3p	MS00003857
hsa-miR-21-5p	MS00009079
hsa-miR-223-3p	MS00003871
hsa-miR-214-3p	MS00031605
hsa-miR-199a-5p	MS00006741
hsa-miR-199a-3p	MS00007602
hsa-miR-195-5p	MS00003703
hsa-miR-150-5p	MS00003577
hsa-miR-145-5p	MS00003528
hsa-miR-130a-3p	MS00003444

Supplementary Table 4: Clinical characteristics of the patients in the Tumor Cancer Genome Atlas datasets (*n* = 373)*

Characteristics	Value
Male (%)	240 (64.3%)
Age (year)	59.7 ± 13.1
Etiology (*n* = 354)	
Alcohol	75 (21.2%)
Hepatitis B	75 (21.2%)
Hepatitis C	32 (9.0%)
Alcohol/Viral hepatitis	39 (11.0%)
Other	28 (7.9%)
Unknown	105 (29.7%)
Tumor grade (*n* = 349)	
G1/2	218 (62.5%)
G3/4	131 (37.5%)
Pathologic stage (*n* = 333)	
Stage I/II	247 (74.2%)
Stage III/IV	86 (25.8%)
MVI	91/286
Mortality	87 (23.3%)

*Clincal information was not available in 19 patients.
MVI, microscopic vascular invasion
